# Chest radiograph classification and severity of suspected COVID-19 by different radiologist groups and attending clinicians: multi-reader, multi-case study

**DOI:** 10.1007/s00330-022-09172-w

**Published:** 2022-10-25

**Authors:** Arjun Nair, Alexander Procter, Steve Halligan, Thomas Parry, Asia Ahmed, Mark Duncan, Magali Taylor, Manil Chouhan, Trevor Gaunt, James Roberts, Niels van Vucht, Alan Campbell, Laura May Davis, Joseph Jacob, Rachel Hubbard, Shankar Kumar, Ammaarah Said, Xinhui Chan, Tim Cutfield, Akish Luintel, Michael Marks, Neil Stone, Sue Mallet

**Affiliations:** 1 Department of Radiology, University College London Hospital, 235 Euston Road, London, NW1 2BU UK; 2 Centre for Medical Imaging, University College London, UCL Centre for Medical Imaging, 2nd Floor Charles Bell House, 43-45 Foley Street, London, W1W 7TS UK; 3 Centre for Medical Image Computing, Department of Computer Science, University College London, 90 High Holborn, Floor 1, London, WC1V 6LJ UK; 4 Department of Tropical and Infectious Diseases, University College London Hospital, 235 Euston Road, London, NW1 2BU UK

**Keywords:** Coronavirus, X-ray, Diagnosis, Observer variation

## Abstract

**Objectives:**

To quantify reader agreement for the British Society of Thoracic Imaging (BSTI) diagnostic and severity classification for COVID-19 on chest radiographs (CXR), in particular agreement for an indeterminate CXR that could instigate CT imaging, from single and paired images.

**Methods:**

Twenty readers (four groups of five individuals)—consultant chest (CCR), general consultant (GCR), and specialist registrar (RSR) radiologists, and infectious diseases clinicians (IDR)—assigned BSTI categories and severity in addition to modified Covid-Radiographic Assessment of Lung Edema Score (Covid-RALES), to 305 CXRs (129 paired; 2 time points) from 176 guideline-defined COVID-19 patients. Percentage agreement with a consensus of two chest radiologists was calculated for (1) categorisation to those needing CT (indeterminate) versus those that did not (classic/probable, non-COVID-19); (2) severity; and (3) severity change on paired CXRs using the two scoring systems.

**Results:**

Agreement with consensus for the indeterminate category was low across all groups (28–37%). Agreement for other BSTI categories was highest for classic/probable for the other three reader groups (66–76%) compared to GCR (49%). Agreement for normal was similar across all radiologists (54–61%) but lower for IDR (31%). Agreement for a severe CXR was lower for GCR (65%), compared to the other three reader groups (84–95%). For all groups, agreement for changes across paired CXRs was modest.

**Conclusion:**

Agreement for the indeterminate BSTI COVID-19 CXR category is low, and generally moderate for the other BSTI categories and for severity change, suggesting that the test, rather than readers, is limited in utility for both deciding disposition and serial monitoring.

**Key Points:**

*• Across different reader groups, agreement for COVID-19 diagnostic categorisation on CXR varies widely.*

*• Agreement varies to a degree that may render CXR alone ineffective for triage, especially for indeterminate cases.*

*• Agreement for serial CXR change is moderate, limiting utility in guiding management.*

**Supplementary Information:**

The online version contains supplementary material available at 10.1007/s00330-022-09172-w.

## Introduction

Coronavirus disease 2019 (COVID-19), caused by the novel severe acute respiratory syndrome coronavirus 2 virus (SARS-CoV-2), became a global pandemic. In the UK, the pandemic caused record deaths and exerted unprecedented strain on the National Health Service (NHS). Facing such overwhelming demand, clinicians must rapidly and accurately categorise patients with suspected COVID-19 into high and low probability and severity. In March 2020, the British Society of Thoracic Imaging (BSTI) and NHS England produced a decision support algorithm to triage suspected COVID-19 patients [1]. This assumed that laboratory diagnosis might not be rapidly or widely available, emphasising clinical assessment and chest radiography (CXR).

CXR therefore assumes a pivotal role, not only in diagnosis but also in the classification and monitoring of severity, which directs clinical decision-making. This includes whether intensive treatment is required (those with “classic severe” disease), along with subsequent chest computed tomography (CT) in those with uncertain diagnosis [2–4] or whose CXR is deteriorating.

Clearly, this requires that CXR interpretation reflects both diagnosis and severity accurately. While immediate interpretation by specialist chest radiologists is desirable, this is unrealistic given demands, and interpretation falls frequently to non-chest radiologists, radiologists in training, or attending clinicians. However, we are unaware of any study that compares agreement and variation between these groups for CXR diagnosis and severity of COVID-19. We aimed to rectify this by performing a multi-case, multi-reader study comparing the interpretation of radiologists (including specialists, non-specialists, and trainees) and non-radiologists to a consensus reference standard, for the CXR diagnosis, severity, and temporal change of COVID-19.

Due to the continued admission of patients to hospital for COVID-19 as the virus becomes another seasonal coronavirus infection, this study has important ongoing relevance to clinical practice.

## Materials and methods

### Study design and ethical approval

We used a multi-reader, multi-case design in this single-centre study. Our institution granted ethical approval for COVID-19-related imaging studies (Integrated Research Application Service reference IRAS 282063). Informed consent was waived as part of the approval.

### Study population and image acquisition

A list of patients aged ≥ 18 years of age consecutively presenting to our emergency department with suspected COVID-19 infection, as per contemporary national and international definitions [5], between 25^th^ February 2020 and 22^nd^ April 2020, who had undergone at least one CXR, was supplied by our infectious diseases clinical team. All CXRs were acquired as computed or digital radiographs, in the anteroposterior (AP) projection using portable X-ray units as per institutional protocol.

### Readers

We recruited four groups of readers (each consisting of five individuals), required to interpret suspected COVID-19 CXR in daily practice, as follows:

- Group 1: Consultant chest radiologists (CCR) (with 7 to 19 years of radiology experience)
- Group 2: Consultant radiologists not specialising in chest radiology (GCR) (with 8–30 years of radiology experience)
- Group 3: Radiology specialist residents in training (RSR) (with 2–5 years of radiology experience
- Group 4: Infectious diseases consultants and senior trainees (IDR) (with no prior radiology experience)

ID clinicians were chosen as a non-radiologist group because, at our institution and others, their daily practice necessitated both triage and subsequent management of COVID-19 patients via their own interpretation of CXR without radiological assistance.

### Case identification, allocation, and consensus standard

Two subspecialist chest radiologists (with 16 and seven years of experience, respectively) first independently assigned BSTI classifications (Table 1) to the CXRs of 266 consecutive eligible patients, unaware of the ultimate diagnosis and all clinical information. Of these, 129 had paired CXRs; that is, they had a second CXR at least 24 h after their presentation CXR. The remaining 137 patients had a single presentation CXR. We included patients with unpaired CXRs as well as paired CXRs to enable us to enrich the study cohort with potential CVCX2 cases, because a high institutional prevalence of COVID-19 during the study period meant that few consecutive cases would be designated “indeterminate” or “normal”. However, evaluating this category is central to understanding downstream management implications for patients. There were 47/137 unpaired CXRs where at least one of the two subspecialist chest radiologists classified the CXR as CVCX2 (indeterminate), and so we used these 47 CXRs to enrich the cohort with CVCX2 cases. The final study cohort comprised 176 patients with 305 CXRs: 129 paired and 47 unpaired.

**Table 1:** BSTI CXR category definitions and interpretation

Code	Category	Main findings and interpretation
CVCX0	Normal	Normal CXR. COVID-19 not excluded- correlate with RT-PCR and clinical suspicion
CVCX1	Classic/probable COVID-19	Lower lobe and peripheral predominant multiple opacities that are usually bilateral
CVCX2	Indeterminate for COVID-19	Does not fit Classic or Non-COVID-19 descriptors- correlate with RT-PCR and clinical suspicion
CVCX3	Non-COVID-19	No typical findings of COVID-19, and other findings suggesting an alternative diagnosis (e.g. pneumothorax, lobar pneumonia, pleural effusion(s), pulmonary oedema)

**Fig. 1:**
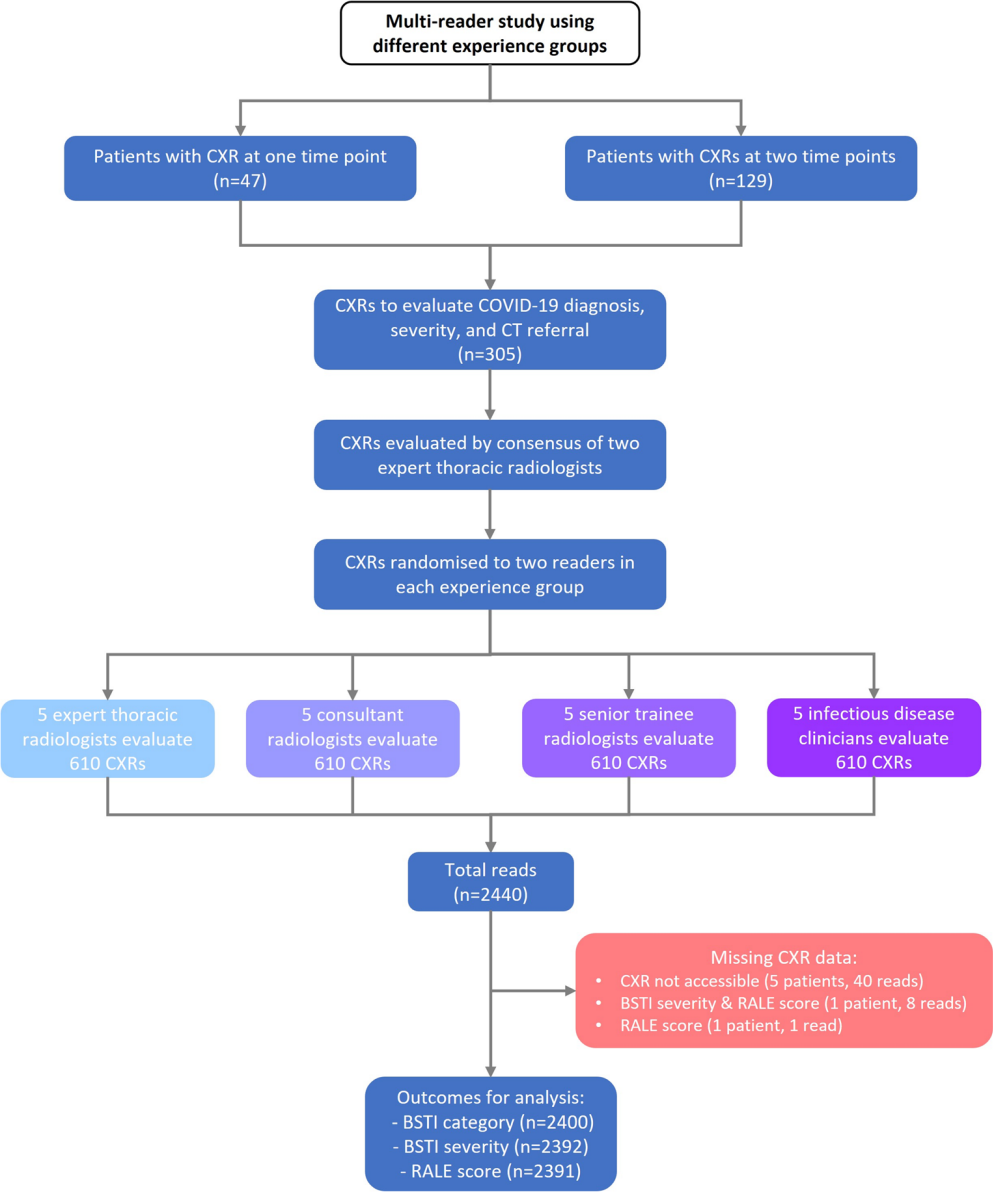
STARD flowchart showing the derivation of CXR reading dataset per reading group

From this cohort of 305 CXRs, five random reading sets were generated, each containing approximately equal numbers of paired and unpaired CXRs (Table 2); each CXR was interpreted by 2 readers from each group. The same reader interpreted both time points for paired CXRs. Minor number variations were due to randomisation. Accordingly, individuals designated Reader 1 (CCR1, GCR1, RSR1, and IDR1) in each group would read the same cases, Reader 2 would read the same, and so on. In this way, 610 reads were generated for each reader group, resulting in 2440 reads overall (Fig. 1). The distribution of the total number of these cases paralleled cumulative COVID-19 referrals to London hospitals over the period under study (Fig. S1).

**Table 2:** Reader allocation of paired and unpaired CXRs for each group

Reader no	No. of paired CXRs	No. of unpaired CXRs	Total
1	104	21	**125**
2	102	21	**123**
3	94	21	**115**
4	102	15	**117**
5	114	16	**130**
TOTAL reads per group	**516**	**94**	**610**

The same two subspecialist chest radiologists assigned an “expert consensus” score to all 305 CXRs at a separate sitting, two months following their original reading to avoid recall bias, blinded to any reader interpretation (including their own).

### Image interpretation

Readers were provided with a refresher presentation explaining BSTI categorisation and severity scoring, with examples. Readers were asked to assume they were reading in a high prevalence “pandemic” clinical scenario, with high pre-test probability, and to categorise incidental findings (e.g. cardiomegaly or minor atelectasis) as CVCX0, and any non-COVID-19 process (e.g. cardiac failure) as CVCX3.

Irrespective of the diagnostic category, we asked readers to classify severity using two scoring systems: the subjective BSTI severity scale (normal, mild, moderate, or severe), and a semiquantitative score (“Covid-RALES”) modified by Wong et al for COVID-19 CXR interpretation from the Radiographic Assessment of Lung Edema (RALE) score [3]. This score quantifies the extent of airspace opacification in each lung (0 = no involvement; 1 = < 25%; 2 = 25–50%; 3 = 50–75%; 4 = > 75% involvement). Thus, the minimum possible score is 0 and the maximum 8. We evaluated this score because it has been assessed by others and is used to assess the severity for clinical trials at our institution.

All cases were assigned a unique anonymised identifier on our institutional Picture Archiving and Communications System (PACS). Readers viewed each CXR unaware of clinical information and any prior or subsequent imaging. Any paired CXRs were therefore read as individual studies, without direct comparison between pairs. Observers evaluated CXRs on displays replicating their normal practice. Thus, radiologists used displays conforming to standards set by the Royal College of Radiologists while ID clinicians used high-definition flat panel liquid crystal display (LCD) monitors used for ward-based clinical image review at our institution.

### Sample size and power calculation

The study was powered to detect a 10% difference between experts and other reader groups for correct detection of CXR reads for CT referral based on indeterminate CXR findings (defined as CVCX2). It was estimated the most experienced group (CCR) would correctly refer 90% of patients to CT. At 80% power, 86 indeterminates would be required to detect a 10% difference in referral to CT using paired proportions, requiring 305 CXRs (176 patients) based on the prevalence of uncertain findings in pre-study reads of CXRs by 2 expert readers > 1 months prior to study reads.

### Statistical analysis

The primary outcome was reader group agreement with expert consensus for an indeterminate CXR which, from the BSTI is the surrogate for CT referral. Indeterminate COVID-19 (CVCX2) is the potential surrogate for triage for CT, but an alternative clinical triage categorisation for CT referral would be to combine “indeterminate” and “normal” BSTI categories (CVCX0 and CVCX2). Therefore, we first calculated the percentage agreement between each reader and the consensus reading for each BSTI diagnostic categorisation. We then also assessed this percentage agreement when the BSTI categorisation was dichotomised to (1) CVCX0 and CVCX2 (i.e. the categories that might still warrant CT if there was sufficiently high clinical suspicion), versus (2) CVCX1 and CVCX3 (i.e. the categories that would probably not warrant CT). We assessed agreement for BSTI severity scoring. All percentage agreements are described with their means and 95% confidence intervals per reader group.

Finally, for paired CXR reads we calculated the number and percentage agreement between each group and the consensus standard for no change, decrease, or increase in (1), the BSTI severity classification and (2), the COVID-RALES.

## Results

### Baseline characteristics

The 176 patients had a median age of 70 years (range 18–99 years); 118 (67%) were male. Due to image processing errors, a CXR was unreadable in one patient without paired imaging and three with, leaving 301 CXRs.

The expert consensus assigned the following BSTI categories: CVCX0 in 97 (32%), CVCX1 in 119 (40%), CVCX2 in 58 (19%), and CVCX3 in 27 (9.0%). Consensus BSTI severity was normal, mild, moderate, or severe in 97 (32%), 93 (31%), 68 (23%), and 27 (14%) respectively. The median consensus COVID-RALES was 2, IQR 0 to 4, range 0 to 8).

### Agreement for indeterminate category (Fig. 2)

Our primary outcome was reader group agreement with expert consensus for indeterminate COVID-19 (CVCX2), reflecting potential triage to CT. The mean agreement for CVCX2 was generally low (28 to 37%). For all reader groups, the main alternative classification for CVCX2 was CVCX1 (“classic” COVID-19), followed by CVCX3 (not COVID-19) (Fig. S2). Even CCR1 and CCR2, who were the two subspecialist readers composing the expert consensus, demonstrated low agreement with their own consensus for CVCX2 (Fig. S3). These data suggest that basing CT referral on CXR interpretation is unreliable, even when interpreted by chest subspecialist radiologists.

**Fig. 2:**
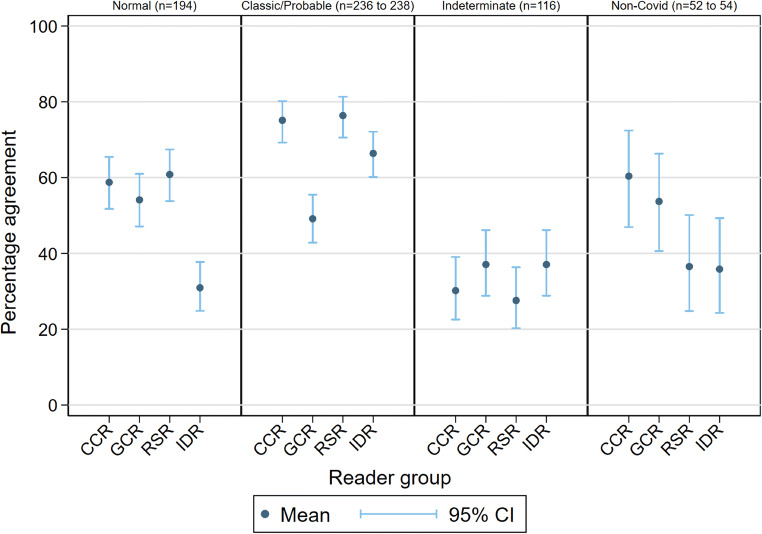
Percentage agreement with consensus for individual BSTI categories for reader groups

An alternative clinical triage categorisation for CT referral would be to combine “indeterminate” and “normal” BSTI categories (CVCX0 and CVCX2), which resulted in higher agreement (CCR 73% (95% CI 68%, 77)% , RSR 75% (71%, 79%), GCR 58% (53%, 62%), and IDR 61% (56%, 65%)).

### Agreement for BSTI categorisation (Table 3 and Fig. 2)

Agreement was highest for CVCX1 (“classic/probable”) for the CCR (75% (69%, 80%)), RSR (76% (71%, 81%)), and IDR (66%(60%, 72%)) groups, but interestingly not for GCR (49% (43%, 55%)), where agreement was comparable to their agreement for CVCX0 and CVCX3 (“non-COVID-19”) (although still higher than their agreement for CVCX2 (“indeterminate”)). When disagreeing with the consensus CVCX1, GCR were most likely to assign CVCX2 (Fig. S1).

**Table 3:** Percentage agreement with consensus for individual BSTI categories for reader groups

Reader group	Agreement for BSTI category (%)			
	CVCX0	CVCX1	CVCX2	CVCX3
CCR	59 (52, 65)	75 (69, 80)	30 (23, 39)	60 (47, 72)
GCR	54 (47, 61)	49 (43, 55)	37 (29, 46)	54 (41, 66)
RSR	61 (54, 67)	76 (71, 81)	28 (20, 36)	37 (25, 50)
IDR	31 (25, 38)	66 (60, 72)	37 (29, 46)	36 (24, 49)

Values given are means (95% lower and upper limits)

Agreement with consensus for CVCX0 (“normal”) was similar for radiologists of all types (mean agreement for CCR, GCR, and RSR of 59%, 54%, and 61% respectively), but lower for IDR (31%). For CVCX3 (not COVID-19), CCR and GCR were generally more likely than RSR and IDR readers to agree with the consensus.

### Agreement for BSTI severity classification (Table 4 and Fig. 3)

Agreement that classification was “severe” was highest for all groups, but lower for GCR (65% (54%, 74%)) than other groups (means of 95% (89%, 98%), 84% (74%, 90%), and 84% (75%, 90%) for CCR, RSR, and IDR respectively). The majority of consensus-graded normal cases were likely to be designated “mild” (Fig. S4).

**Table 4:** Percentage agreement with consensus for BSTI severity classification for reader groups

Reader group	Agreement for BSTI Severity (%)			
	Normal	Mild	Moderate	Severe
CCR	59 (52, 65)	52 (45, 59)	55 (46, 63)	(95 (89, 98)
GCR	55 (48, 62)	65 (58, 72)	69 (61, 76)	64 (54, 74)
RSR	62 (55, 69)	58 (51, 65)	47 (39, 55)	47 (39, 55)
IDR	30 (24, 37)	45 (38, 52)	51 (43, 59)	84 (75, 90)

Values given are means (95% lower and upper limits)

**Fig. 3:**
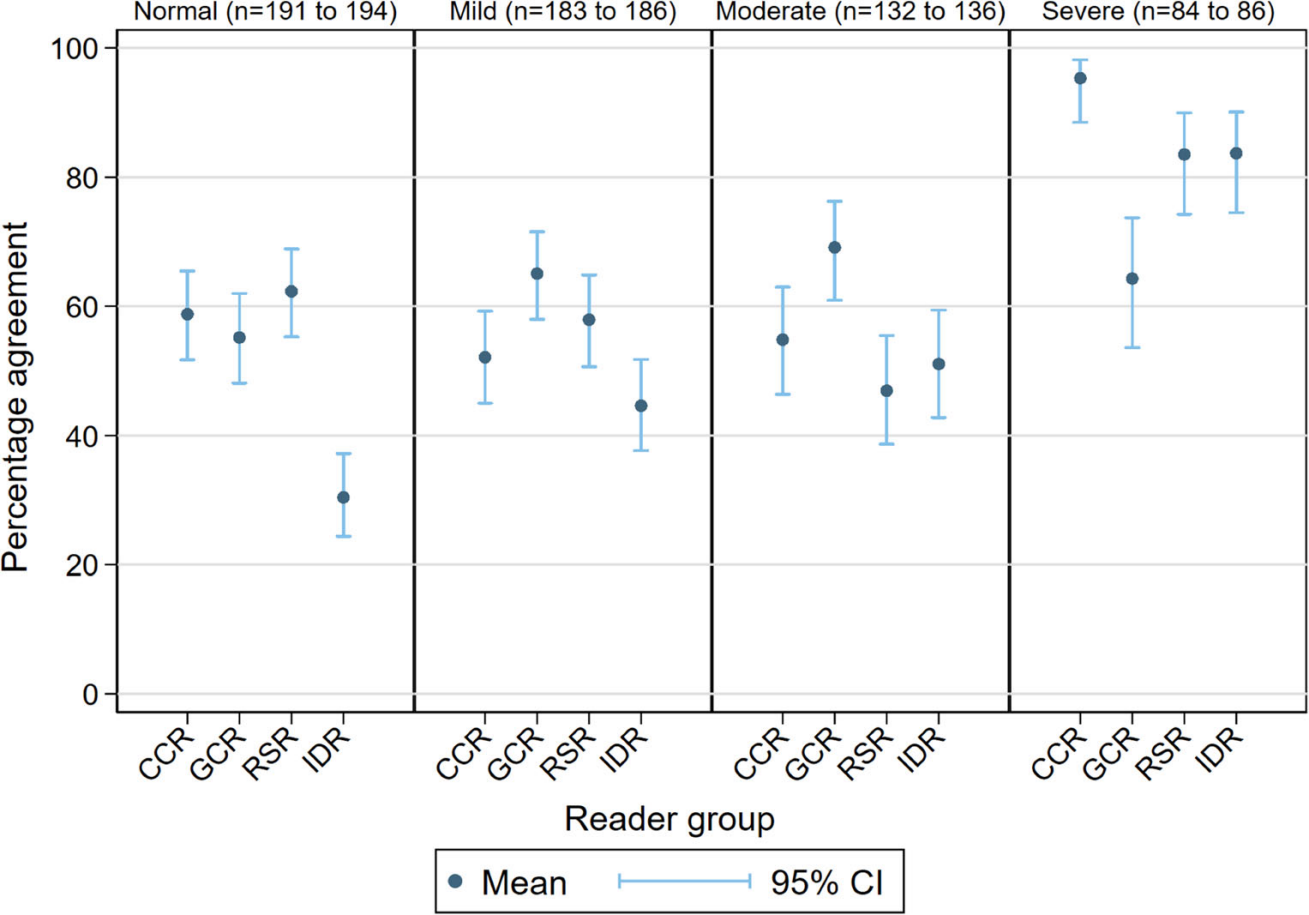
Percentage agreement with consensus for BSTI severity classification for reader groups

### Agreement for change on CXRs (Table 5 and Fig. 4)

The expert consensus reference found that the majority of BSTI severity scores did not change where paired CXR examinations were separated by just one or two days. Using the BSTI severity classification, the highest agreement with consensus across all groups was for “no change”, with percentage agreement of 66%, 61%, 44%, and 48% for CCR, GCR, RSR, and IDR respectively.

**Table 5:** Percentage agreement for change in BSTI severity classification and Covid-RALES for reader groups

Reader Group	Serial CXR change					
	BSTI severity			Covid-RALES		
	No change	Decrease	Increase	No change	Decrease	Increase
CCR	85 (66)	1 (6)	31 (29)	42 (50)	9 (28)	79 (57)
GCR	77 (61)	3 (17)	36 (33)	37 (44)	9 (28)	80 (59)
RSR	56 (44)	4 (25)	41 (38)	29 (35)	11 (37)	81 (59)
IDR	62 (48)	7 (39)	31 (29)	29 (35)	13 (41)	64 (47)

Figures given are numbers (percentages)

**Fig. 4:**
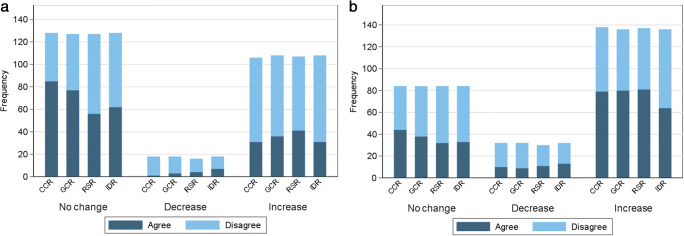
Frequency charts showing agreement with consensus for score change using the BSTI severity classification (**a**) and the Covid-RALES for reader groups (**b**)

In contrast, when using Covid-RALES, the highest agreement with consensus across all groups was for an “increased score”, with percentage agreement of 57%, 59%, 59%, and 47% for CCR, GCR, RSR, and IDR respectively. This most likely reflects the larger number of individual categories assigned by Covid-RALES.

## Discussion

Thus far, studies of CXR for COVID-19 have either reported its diagnostic accuracy [6, 7], implications of CXR severity assessment using various scores [4, 8–10], or quantification using computer vision techniques [11–13]. Inter-observer agreement for categorisation of COVID-19 CXRs, including for the BSTI classification (but not BSTI severity) has been assessed amongst consultant radiologists [14], while inter-observer differences according to radiologist experience have been described [15, 16]. Notably, in a case-control study, Hare et al compared the agreement for the BSTI classification amongst seven consultant radiologists, including two fellowship-trained chest radiologists (with the latter providing the reference standard). They found that only fair agreement was obtained for the CVCX2 category *κ* = 0.23), and “non-COVID-19” (*κ* = 0.37) categories, but that combining the scores of the CVCX2 and CVCX3 scores improved inter-observer agreement (*κ = *0.58) [14]. A recent study compared the sensitivity and specificity (but not agreement) of using the “classic/probable” BSTI category for COVID-19 diagnosis between Emergency Department clinicians and radiologists (both of various grades), based on a retrospective review of their classifications [17].

Our study differs in that it pivots around three potential clinical scenarios that use the CXR to manage suspected COVID-19. Using a prospective multi-reader, multi-case design, we determined reader agreement for four clinical groups who are tasked with CXR interpretation in daily practice and compared these to a consensus reference standard. Firstly, we evaluated reader agreement when using CXR to triage patients for CT when CXR imaging is insufficient to diagnose COVID-19. Secondly, we examined agreement for disease severity using two scores (BSTI and RALES). Thirdly, we investigated whether paired CXRs could monitor any change in severity.

When CXR was used to identify which patients need CT, based on our pre-specified BSTI category of an indeterminate interpretation, agreement with our consensus was low (28 to 37%) or moderate (58 to 75%) respectively. All four reader groups had a similar agreement to the consensus for identifying indeterminates, indicating that the level or specialism or radiologist expertise did not enhance agreement. When combining indeterminates with normal, GCR and IDR groups had lower agreement because the GCR group assigned more indeterminates as non-COVID-19, whereas the IDR group assigned more to classic/probable COVID-19.

Similar (albeit modest) agreement for the “normal” category amongst radiologists of all grades and types suggests that these factors are not influential when assigning this category. Radiologists seemed willing to consider many CXRs normal despite assuming a high prevalence setting. Reassuringly, this suggests that patient disposition, if based on normal CXR interpretation, is unlikely to vary much depending on the category of radiologist. Conversely, the lower agreement of ID clinicians for a normal CXR suggests an inclination to overall abnormal, since they classified normal CXRs as mostly “indeterminate” but also “classic/probable” COVID-19. We speculate that the contemporary pandemic clinical experience of ID clinicians made it difficult for them to consider a CXR normal, even when deprived of supporting clinical information.

In contrast, general consultant radiologists were less inclined to assign the “classic/probable” category, predominantly favouring the indeterminate category. Our results are somewhat at odds with Hare et al [14], who found substantial agreement for the CVCX1 category amongst seven consultant radiologists. Reasons underpinning the reticence to assign this category (even in a high prevalence setting) are difficult to intuit but may be partly attributable to a desire to adhere to strict definitions for this category, and thus maintain specificity.

Severity scores can quantify disease fluctuations that influence patient management, have prognostic implications [8–10], and may also be employed in clinical trials. However, this is only possible if scores are reliable, which is reflected by reader agreement regarding both value and change. For our second and third clinical scenarios for CXR, we also found that assessment of severity and change, and therefore of CXR severity itself, varied between reader groups and readers using either severity scoring system, but in different ways. It is probably unsurprising that agreement for no change in BSTI severity was highest for all reader groups, given that the four-grade nature of that classification is less likely to detect subtle change. In contrast, the finer gradation of Covid-RALES allows smaller severity increments to be captured more readily. A higher number of categories also encourages disagreement; despite this, agreement was modest.

We wished to examine CXR utility in a real-world clinical setting using consecutive patients presenting to our emergency department with suspected COVID-19 infection. Our findings are important because they examine clear clinical roles for CXR beyond a purely binary diagnosis of COVID-19 versus non-COVID-19. Rather, we examined the CXR as an aid to clinical decision-making and as an adjunct to clinical and molecular testing. CXR has moderate pooled sensitivity and specificity for COVID-19 (81% and 72% respectively) [18] and, in the context of other clinical and diagnostic tests [19], such diagnostic accuracy could be considered favourable. Although thoracic CT has a higher sensitivity for diagnosing COVID-19 [18], CXR has been used and investigated in this triage role both in the UK and internationally [20]. However, our results do have important implications when using CXR for diagnosis because interpretation appears susceptible to substantial inter-reader variation. Investigating reader variability will also be crucial for development, training, and evaluation of artificial intelligence algorithms to diagnose COVID-19, such as that now underway using the National COVID-19 Chest Imaging Database (NCCID) [21].

Our study has limitations. ID clinicians, as the first clinicians to assess potential COVID-19 cases, were the only group of non-radiologist clinicians evaluated. While we would wish to evaluate the emergency department and general medical colleagues also, this proved impractical. However, we have no a priori expectation that these would perform any differently. Our reference standard interpretation used two subspecialist chest radiologists; like any subjective standard, ours is imperfect, but with precedent [14]. We point out that our data around variability of reader classifications are robust regardless of the reference standard (see data in supplementary Figs. S2 and S4). Arguably, we disadvantaged ID clinicians by requiring them to interpret CXRs using LCD monitors, but this reflects normal clinical practice. It is possible that readers may have focussed on BSTI diagnostic categories in isolation, rather than considering the implications of how their categorisation would be used to decide patient management but, again, this reflects normal practice (since radiologists do not determine management). Readers did not compare serial CXRs directly, but read them in isolation: We note a potential role for monitoring disease progression when serial CXRs are viewed simultaneously, but this outcome would require assessment by other studies.

In conclusion, across a diverse group of clinicians, agreement for BSTI diagnostic categorisation for COVID-19 CXR classification varies widely for many categories, and to such a degree that may render CXR ineffective for triage using such categories. Agreement for serial change over time was also moderate, underscoring the need for cautious interpretation of changes in severity scores if using these to guide management and predict outcome, when these scores have been assigned to serial CXRs read in isolation.

## Supplementary information

ESM 1 (DOCX 1216 kb)

## Abbreviations

BSTI: British Society of Thoracic Imaging
CCR: Consultant chest radiologists
Covid-RALES: Radiographic Assessment of Lung Edema (RALE) score, modified for COVID-19 interpretation
CVCX: BSTI COVID-19 chest radiograph category
CXR: Chest radiograph
GCR: General consultant radiologists
IDR: Infectious diseases consultants and senior trainees
NHS: National Health Service
RSR: Radiology specialist residents in training

## Declarations

### Guarantor

The scientific guarantor of this publication is Arjun Nair (arjun.nair1@nhs.net)

### Statistics and biometry

One of the authors has significant statistical expertise- Professor Sue Mallet is a Professor in diagnostic and prognostic medical statistics, specialising in clinical trial design, methodology, and systematic reviews. She is a senior author of the paper.

### Methodology

• retrospective

• observational

• performed at one institution
