# Supplementary material for: Chest radiograph classification and severity of suspected COVID-19 by different radiologist groups and attending clinicians: multi-reader, multi-case study
Source: Eur Radiol. 2022 Oct 25;33(3):2096–104. doi: 10.1007/s00330-022-09172-w (PMC9592875; doi:10.1007/s00330-022-09172-w)

# Supplementary Figures

Figure S1. Recruitment of cases relative to COVID hospital referrals to London hospitals


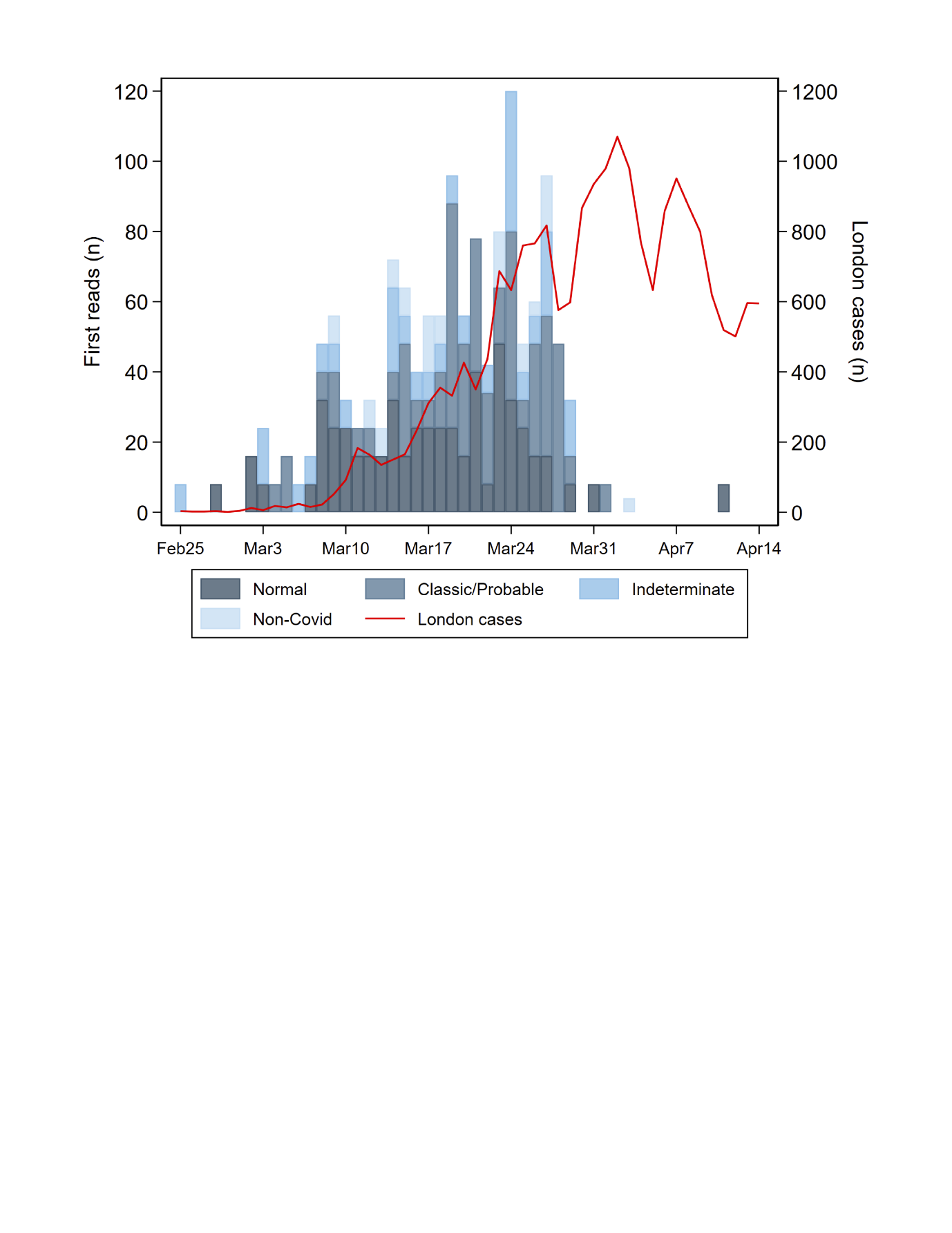


Figure S2. Percentage agreement with consensus for individual BSTI categories, sub-divided into all classifications assigned for a given consensus category.


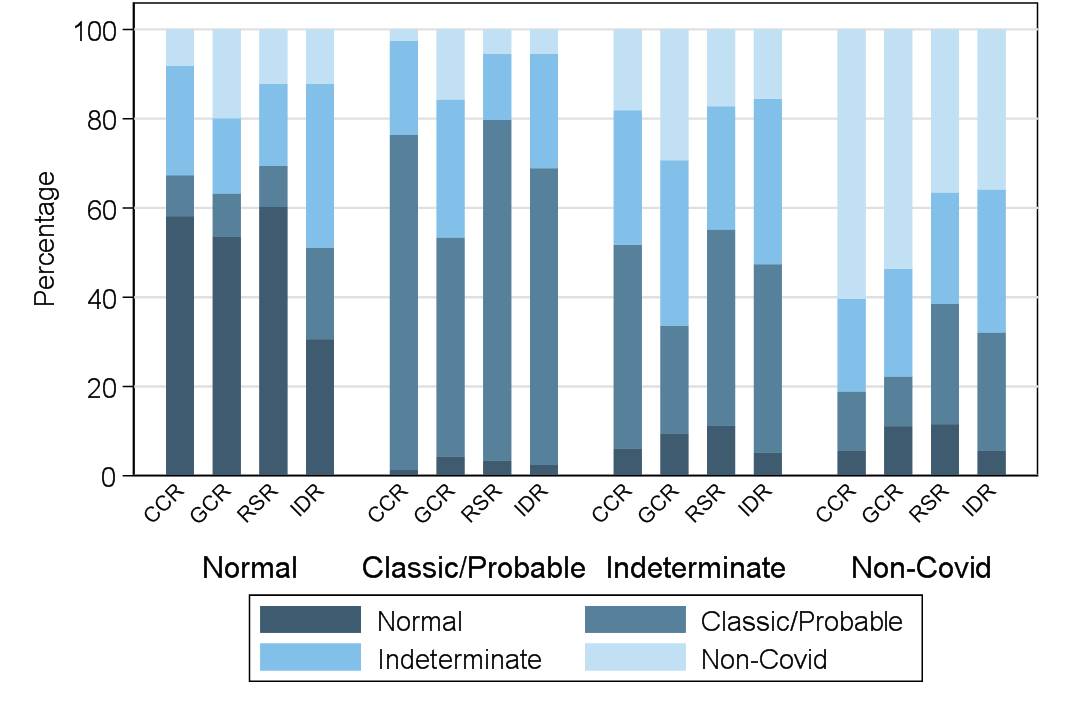


Figure S3. Percentage agreement with consensus for individual BSTI categories for each individual reader.


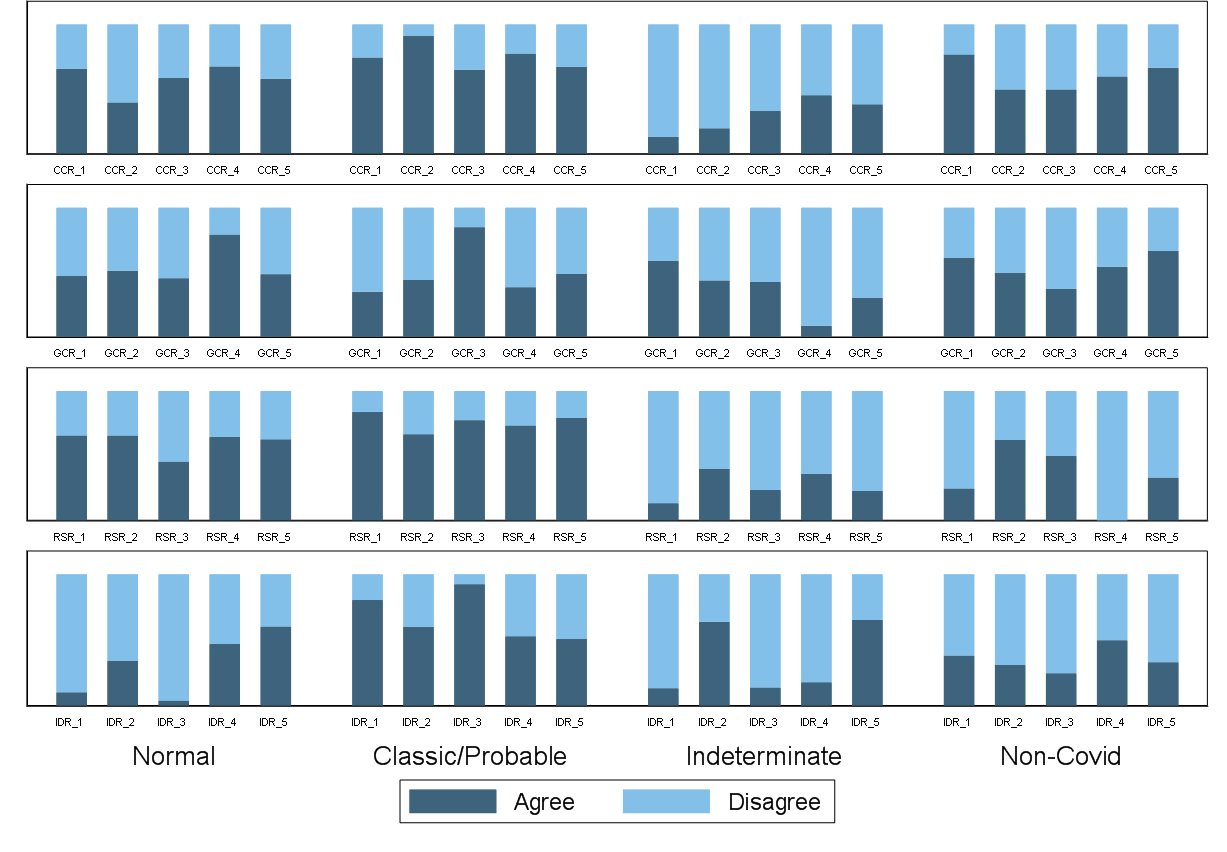


Figure S4. Percentage agreement with consensus for BSTI severity classification, sub-divided into all classifications assigned for a given consensus severity classification.


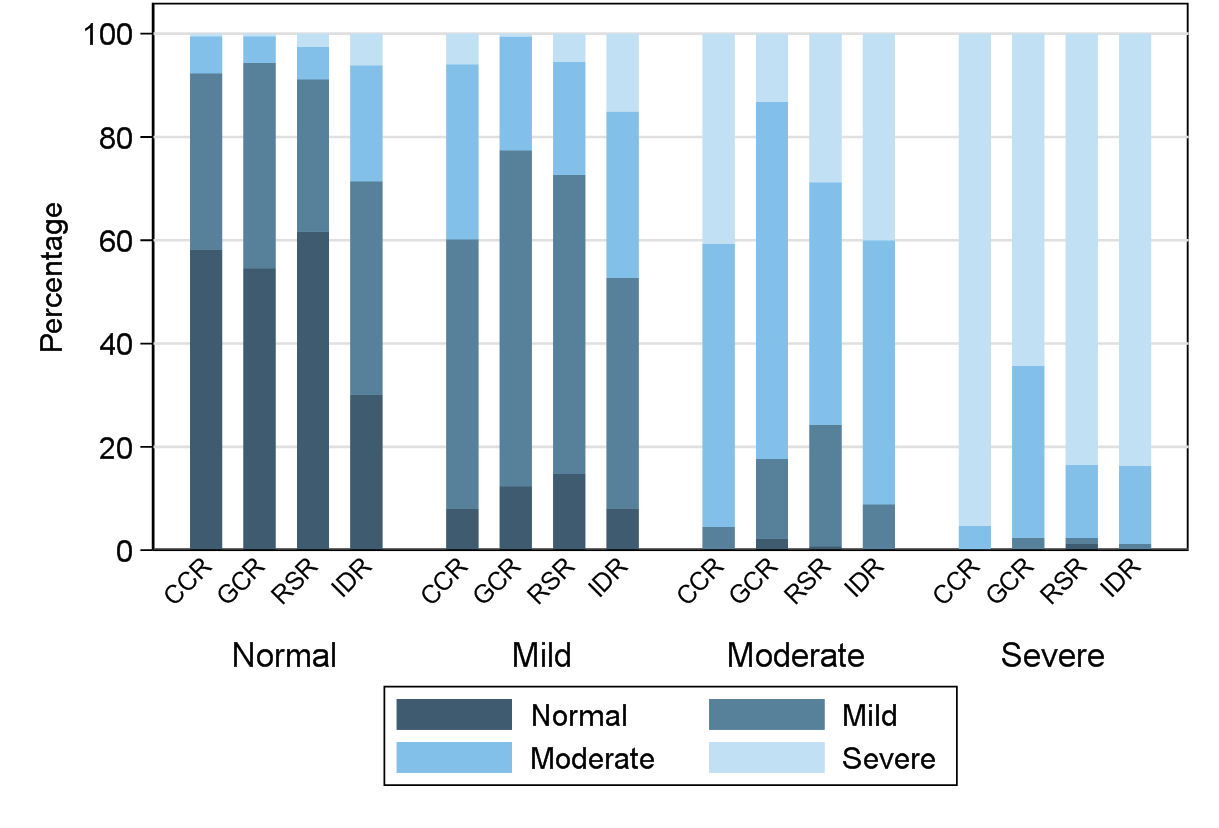

Supplement: Supplementary file 1 — (DOCX 1216 kb) [file 330_2022_9172_MOESM1_ESM.docx]
